# Effects of bracing on pulmonary function in adolescent idiopathic scoliosis: a systematic review

**DOI:** 10.1186/s12891-026-10241-0

**Published:** 2026-07-22

**Authors:** Yuen Tong Law, Lok Sze Hon, Ka Hei Lam, Oi Kwan Lam, Kit Chi Yeung, Sharon Man Ha Tsang, Adam Yiu Chung Lau

**Affiliations:** 1 SH Ho Scoliosis Research Laboratory, Joint Scoliosis Research Centre of the Chinese University of Hong Kong and Nanjing University, Department of Orthopaedics and Traumatology, The Chinese University of Hong Kong, Prince of Wales Hospital, Hong Kong SAR, China; 2 Physiotherapy Department, Prince of Wales Hospital, Hong Kong SAR, China; 3 Department of Rehabilitation Sciences, The Hong Kong Polytechnic University, Hong Kong SAR, China

**Keywords:** Adolescent idiopathic scoliosis, Bracing, Pulmonary functions, Review

## Abstract

**Background content:**

Adolescent idiopathic scoliosis (AIS) is the most common form of 3-dimensional pediatric spinal deformity, characterized by abnormal lateral displacement and rotation. Full-time rigid bracing is often prescribed to prevent curve progression during skeletal growth. Directed force is applied to the apex via the rib cage to achieve satisfactory in-brace correction. The short- and long-term effects of bracing on the pulmonary function remain unclear.

**Purpose:**

To study the effects of bracing on the pulmonary function of AIS patients.

**Study design:**

Systematic review.

**Outcome measures:**

FEV1, FVC, VC and FEV1/FVC ratio.

**Methods:**

Following the PRISMA guidelines, PubMed, Cochrane Library, Scopus, EBSCOhost and PEDro were analyzed from inception to 15 October 2025, searching keywords of “AIS”, “bracing” and “Pulmonary function”. Original English research reporting outcomes on AIS patients with bracing as a core intervention and measurements of pulmonary function parameters are included, while research including other interventions are excluded. 15 studies were selected, totaling 578 patients and 731 controls. ROBINS-I (Risk Of Bias In Non-randomized Studies -of Interventions) was used to assess risk of bias. Data extraction is performed on the papers, while the quality appraisal was done using the MINORS methodology score. Due to the heterogeneity of bracing regimen and pulmonary function outcomes adopted across included studies, a structured narrative synthesis approach instead of the meta-analysis was taken to summarize the findings.

**Results:**

Short-term: Bracing is associated with a reduction in pulmonary function; however, there is no change in FEV1/FVC ratio.

Long-term effects: AIS patients show a decrease in pulmonary function when the brace on in both the first and eighth month of bracing compared to brace off. Adolescents who received bracing treatment showed an improvement in pulmonary function 25 years after treatment compared to those untreated; however, their respiratory function remains worse than that of those without scoliosis.

**Conclusions:**

The principle and mechanism of bracing pose a restrictive pattern on patients with AIS. There is an immediate decrease in the pulmonary function when the patients are braced. The decline in pulmonary function both in and out-brace increases when the patient is braced over a prolonged period of time (1 month vs 8 months). However, in longitudinal follow-up of up to 25 years post-treatment revealed that the pulmonary functions (both FEV1 and VC) improved significantly compared to pre-bracing (*p* < 0.001, with medium standard mean difference of 0.4 and 0.614 respectively), suggesting possible long-term benefits of bracing.

## Introduction

Adolescent idiopathic scoliosis (AIS) is the most widely seen form of spinal deformity in the pediatric population between the ages of 10 to 18. AIS is a three-dimensional deformity of the spine with an undefined etiology that manifests predominantly with abnormal lateral spinal curvature, combined with vertebral rotation, with a prevalence of approximately 0.47% to 5.2% globally [1–3]. Those with coronal curvatures larger than 25 degrees but smaller than 40 to 45 degrees measured by the Cobb angle are considered ideal candidates for bracing [4]. Bracing is an indispensable non-surgical intervention for managing AIS. Full-time rigid bracing methods are commonly deployed, aiming to control the progression of scoliosis to prevent surgery [5]. Full-time bracing protocols are recommended for their efficiency in controlling the curve progression. Studies have found that increased hours of brace wear correlate significantly with higher treatment success rates [6, 7]. Boston, Wilmington, Cheneau, Miami, Rosenberg, and Sforzesco braces are widely used, requiring the patients to wear the tailor-made brace for 20–24 h per day. Nighttime rigid bracing, such as the Charleston Brace, would require patients to wear them for 8–12 h during sleep [8].

Results are divided on whether bracing would affect a person’s respiratory function. However, concern had been raised related to the potential unfavorable impacts of full-time bracing on the respiratory functions amongst those with AIS for an average duration from two to four years until their skeletal maturation and the end of bone growth [6]. Scoliosis would cause impaired respiratory function by restricting rib movements and chest wall expansion, which ultimately impairs the efficiency of the respiratory muscle functions. Furthermore, scoliosis would reduce chest wall expansion and lung compliance, hence impeding respiratory mechanisms and functions [3]. Some believe that the rigid braces could correct scoliosis and subsequently improve respiratory functions; on the contrary, others argue that the rigid braces would limit the natural chest wall expansion extrinsically. The extrinsic restriction may have a potential negative impact on the parenchymal lung tissue compliance, similar to the etiology of the restrictive lung condition. The critic group advocated that bracing would reduce lung expansion and contribute to an overall increase in chest stiffness, affecting respiratory function, particularly the lung volume performance [9–12]. As there is no systematic review nor meta-analysis currently present on the topic, a review is required to draw a conclusion. To better understand this inconsistency identified from the present literature, this review aimed to systematically review and synthesize the available evidence on whether full-time bracing (compared to those unbraced or pre-braced) could improve the respiratory function of patients with AIS using the major volumetric measures of the respiratory functions, namely Forced Expiratory Volume in 1 Second (FEV1) and Forced Vital Capacity (FVC), Vital Capacity (VC) and the ratio between Forced Expiratory Volume in 1 Second and Forced Vital Capacity (FEV1/FVC ratio) which defines the short-term effect, as well as the long-term effects that follow.

## Materials and methods

### Search strategies

A comprehensive search of currently available literature was conducted in accordance with the Preferred Reporting Items for Systematic Reviews and Meta-Analyses (PRISMA) checklist [13]. The search was implemented in a total of five electronic databases, namely PubMed, Cochrane Library, Scopus, EBSCOhost and PEDro from inception to 15 October 2025. The search strategy for each database included a combination of keywords and terms using the PICO framework. The full strategy for all databases was as follows: (idiopathic scoliosis OR adolescent idiopathic scoliosis) AND (bracing OR brace) AND (respiratory function OR respiratory muscle OR inspiratory muscle OR pulmonary function OR ventilatory function). Only English papers were evaluated. No limit was applied to the publication year. Relevant titles, abstracts and keywords were searched for in Cochrane Library and Scopus, EBSCOhost, and PEDro, while only titles and abstracts were searched within PubMed. The number of search results in PubMed, Cochrane Library, Scopus, EBSCOhost, and PEDro was 79, 27, 25, 18 and 0, respectively. The data was analyzed and the study is registered in PROSPERO (PROSPERO 2025 CRD420251163159, 10 Oct 2025).

### Article screening process and the selection of studies

**Fig. 1:**
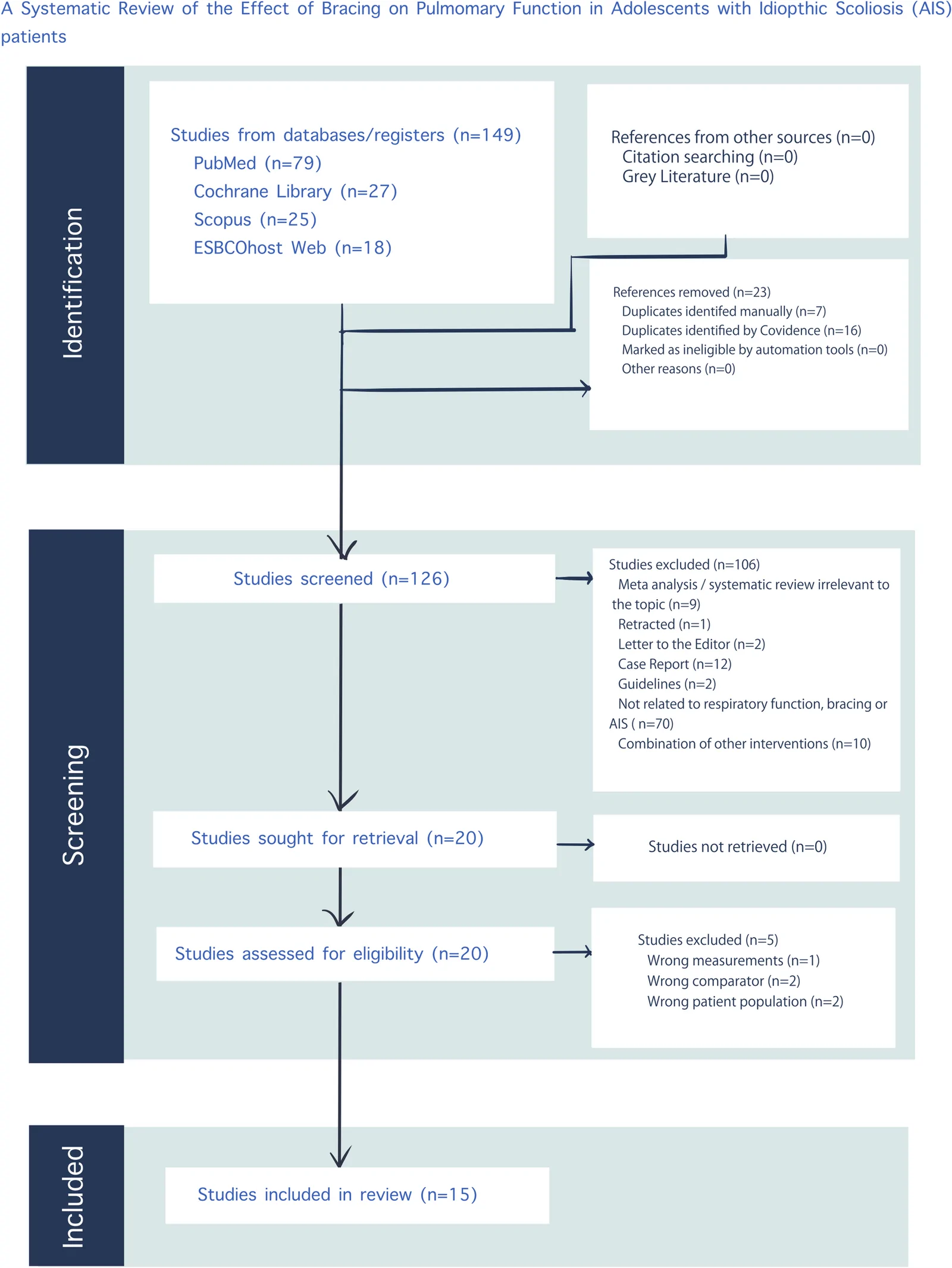
PRISMA flow diagram

All related academic papers on the effect of bracing on pulmonary function in AIS patients were reviewed by four independent reviewers (L.Y.T., H.L.S., L.K.H., L.O.K.). Duplicated records were removed and excluded before screening. Of the 149 entries, 23 were duplicated. The four reviewers then independently filtered the remaining search results by title and abstract. Out of 126 articles, 106 were excluded because they were either retracted, did not meet the inclusion criteria (Table 1), or did not involve bracing or the measurement of pulmonary or respiratory function. If an abstract was unavailable, the decision to include or exclude an article was based on its title. In cases of uncertainty, the reviewers discussed the related articles as a group and achieved a consensus decision accordingly. The 30 articles retained were obtained in full texts, and researchers evaluated if the papers met the inclusion criteria of this review. Figure 1 concludes the study selection process (Table 2).

**Table 1:** Study selection criteria

Inclusion Criteria	Exclusion Criteria
English language	Case report
Original research	Review
Reported outcomes on subjects with adolescent idiopathic scoliosis (AIS)	Medical Guidelines
Including bracing as the core intervention	Letter to the editor
Prospective cohort, retrospective cohort, cross-sectional, case control, randomized controlled trials and case series	Combination with other interventions (e.g. exercise, Chinese medicine and acupuncture)
Any pulmonary function parameter measured, e.g Forced expiratory volume (FEV1) Forced vital capacity (FVC) Vital capacity (VC) Ratio between forced expiratory volume/forced vital capacity (FEV1/FVC ratio) Others including but not limited to oxygen consumption, questionnaires or scales for measuring respiratory functions

**Table 2:** Reasons for exclusion after full text screening

Study	Reason for exclusion
Physical work capacity in adolescent patient with mild idiopathic scoliosis (DiRocco et al. 1983) [14]	It focuses on mild idiopathic scoliosis and Milwaukee brace, but more importantly the outcome domain is cardiorespiratory/exercise capacity, not spirometric respiratory function. Key findings such as FVC, FEV1 and VC are absent
Short-term effects of bracing on exercise performance in mild idiopathic thoracic scoliosis (Ferrari et al. 1997) [15]	Pulmonary key findings such as FVC, FEV1 and VC are absent. Instead, this review focuses on parameters such as heart rate, CO_2_ output and O_2_ uptake
Health-related quality of life and functional outcomes in patients with congenital or juvenile idiopathic scoliosis after an average follow up of 25 years: a cohort study (Heemskerk et al. 2024) [16]	The pulmonary parameters were not measured. Instead, this is a study that concludes the functional outcome and whether the physical activities of the patients were affected
Pulmonary function in patients with idiopathic scoliosis 40 years after diagnosis (Ragborg et al. 2024) [17]	Not relevant. Although the patients had adolescent idiopathic scoliosis, and 50 of them underwent brace treatment, they were not separately compared from the other groups who underwent other therapies. Also, there is no pre and post brace comparison
Effect of the Milwaukee brace on vital and ventilatory capacity of scoliotic patients (Sevastikoglou et al. 1976) [18]	Partially relevant; the data encompasses both AIS patients and childhood idiopathic scoliosis. The respiratory function data is combined so we could not know for sure the effectiveness of the Milwaukee brace of AIS patients

Fifteen non-randomized studies were finally included in this review. ROBINS-I is used to assess the risk of bias [19]. The study quality was assessed using the MINORS methodology score on a 12-item form [20]. 16 points is the maximum score for the non-comparative studies (8 items) and 24 points is the maximum score for comparative studies(12 items). Table 5 and 6 show the results of the bias and quality assessment respectively. Finally, a systematic review is planned, which is done by determining the question and the eligibility criteria, selecting relevant studies, assessing risk of bias and extracting information from the studies, summarizing and drawing the conclusion [21].

### Data extraction

A data extraction form (Table 3) was created and filled in during the progress of full-text analysis. It was used to record data relevant to this review, including author(s), year, intervention, method and design of study population inclusion and exclusion criteria, types of braces, population baseline characteristics of age, Cobb angle, thoracic or lumbar Cobb angle, duration of bracing per day, length of bracing in months/years, time windows and pulmonary function parameter. Table 4 details the sample size, which includes the braced sample size, the number of controls and the number of irrelevant data and withdrawals.

**Table 3:** Characteristics of included studies

Study ID	Intervention	Methods: Design	Population: Inclusion criteria	Population: Exclusion criteria	Types of Brace	Population Baseline Characteristics Overall (Bracing/control): Mean age (years) ± SD	Population Baseline Characteristics Overall (Braced/Control): Cobb angle ± SD
Ran et al. 2016 [10]	Bracing(brace treatment group)/Control (without brace treatment group)	Case–control study	(1) A diagnosis of AIS according to Scoliosis Research Society; (2) Undergoing operations with preoperative brace treatment or not; and (3) Had a complete record of demographic, preoperative radiological and pulmonary function data	Other related disorders/spine anomalies	Milwaukee Brace/Boston brace	(12.5 ± 1.4/12.7 ± 1.6) (*n* = 60/177)	(Coronal) (53.7 ± 13/53.4 ± 12.7) (*n* = 60/177)
Lin et al. 2022 [22]	Bracing (off brace)/Control (the rest without a history of preoperative Boston Brace treatment))	Prospective cohort study	Diagnosed with AIS and before surgery	Scoliosis associated with the presence of any congenital heart defect that may affect cardiac function, for example, the presence of ventricular septal defect, patent ductus arteriosus, or tetralogy of Fallot; primary pulmonary diseases; history of cardiopulmonary surgery; history of smoking; and refusal to enroll in the study	Boston brace	14.38 ± 1.69 (*n* = 168)	NR
Yurt et al. 2021 [12]	Bracing(Braced/without brace (18 h off brace before measurement))	Prospective case series	Aged between 10–16 years, having a primary thoracic curve and a Cobb angle ranging from 20◦ to 45◦ were included	Participated in a regular exercise program or sport, had worn a brace earlier underwent spinal surgery had a respiratory disease and smoked	TLSO brace, possibly Boston brace (not specified)	13.2 ± 1.6 (*n* = 15)	Out-brace 25.8 ± 7.7 In-brace 18.3 ± 6.1 (*n* = 15)
Refsum et al. 1990 [23]	Bracing (Before bracing/with brace)	Prospective case series	Girls aged 10–16 years with progressive, previously untreated idiopathic scoliosis	NR	Boston thoracic brace	13.7 (*n* = 49)	32.4, range 19–60 (*n* = 49)
DiMaria et al. 2021 [24]	Bracing (Resting breathing/5 min after wearing Sforesco brace)	Prospective observational study with a pre-post design	1) Confirmed diagnosis of moderate AIS (Cobb angle of 21◦ to 35◦ for the primary curve) (2) A thoracic or thoracolumbar primary curve (3) Skeletal immaturity with growth cartilage visible on pre-treatment radiographs (Risser score < 5); (4) Age of 11 to 16 years; and (5) Ongoing treatment with the Sforzesco brace	(1) scoliosis due to other spine disorders, or linked to known causes (2) present or past smoking habit (3) presence of cardio-respiratory, pulmonary and/or pleural abnormalities (4) upper or lower respiratory tract infections in the 6 week period before the study day	Sforzesco Brace	13.27 ± 1.56 (*n* = 11)	26 ± 4.49 (*n* = 11)
Yu et al. 2013 [25]	Bracing(underwent brace treatment before operation)/Control(Without brace treatment before operation)	Retrospective cohort study	Female AIS patients 10–18 years old	NR	Milwaukee Brace/Boston brace	14.4, range 10–18 (*n* = 270)	51.4, range 35–105 (*n* = 270)
Yagci et al. 2019 [11]	Bracing (Unbraced/1 months after bracing, with brace)	Prospective case series	Having a primary thoracic curve, moderate scoliosis (a Cobb angle of 20°–45° for the primary curve), and a bracing indication	NR	TLSO brace, possibly Boston brace (not specified)	14.5 ± 1.5 (*n* = 27)	NR
Katsaris et al. 1999 [26]	Bracing(without the Boston Brace/Immediately after wearing brace)	Prospective case series	(1) Mild IS (2) Adolescents	(1) Smoker (2) Cardiorespiratory problems (3) Treated with either brace/spinal fusion (4) Congenital or neuromuscular origins of scoliosis	Boston brace	14.1 (*n* = 15)	1.67 (*n* = 15)
Pehrsson et al. 2001 [27]	Bracing(Before treatment/Present follow up/Control group without Scoliosis)	Longitudinal Observational follow-up study	(1) Adolescent idiopathic scoliosis (onset of scoliosis 10–16 years according to Scoliosis Research Society); (2) Treatment either with surgery or brace; (3) Treatment started between 1968 and 1976; (4) Treatment completed before the age of 21 years; (5) Present follow-up performed at least 20 years after completion of treatment	NR	Milwaukee Brace/Boston brace	For brace treated, age of start treatment 14.3 ± 1.4 (range 10.9–18.4) age at completed brace treatment 17.1 ± 1.2 (14.0–20.9) age at present follow up 39.3 ± 2.3 (34.4–45.4) (*n* = 110)	For brace treated, angle at the start of treatment 33 ± 10 (range 12–60) present follow up 38 ± 15 (5–71) (*n* = 110)
Kennedy et al. 1989 [28]	Bracing (Not braced/Immediately after bracing)	Prospective case series	Orthopaedic bracing programme at the Royal Children's Hospital	NR	Milwaukee Brace/Boston brace	13.5 ± 1.12 (*n* = 13)	29 ± 9.13 (*n* = 13)
Danielsson et al. 2015 [29]	Bracing (off brace)/Control (without scoliosis)	Longitudinal Observational follow-up study	1) A diagnosis of idiopathic scoliosis made before the age of 10 years and no other related disorders/spine anomalies 2) Treatment completed no later than at maturity with either a brace (at least 6 months) or surgery 3) Start of treatment between 1966 and 1992, and 4) At least 10 years since maturity or surgical procedure	NR	Milwaukee Brace/Boston brace	10.7 ± 2.8 (*n* = 68)	28.9 ± 8.4 (range 10.0–47.0) (*n* = 68)
Kennedy et al. 1987 [30]	Bracing (Not braced, brace on)	Prospective case series	(1) Idiopathic thoracic scoliosis attending an orthopedic clinic (2) Bracing (Milwaukee or Boston Brace) (3) Normal lung volume	Cardiopulmonary systems impairment	Milwaukee Brace/Boston brace	13.3 (*n* = 33)	29 (*n* = 33)
Korovessis et al. 1996 [31]	Bracing (baseline without brace, baseline with brace, 24 months later with brace, 24 months later without brace)	Prospective case series	Adolescents with previously untreated mild idiopathic scoliosis Recommended for treatment with the Boston Brace	Significant pulmonary insufficiency at rest	Boston Brace	13.6 ± 1.8 (*n* = 36)	NR
León-Morillas et al. 2021 [32]	Bracing (orthotic brace/no intervention)	Retrospective case series	Cobb angle greater than 10 degrees undergoing Conservative treatment or no intervention	Neuromuscular problems and surgical treatment	Cheneau brace	13.5 ± 2.12 (14 ± 1.41/13.29 ± 0.70) (*n* = 11, 4, 7)	16.3 ± 7.19 (23.25 ± 9.60/12.7 ± 2.96) (*n* = 11, 4, 7)
Margonato et al. 2005 [33]	Bracing (pre bracing, after 1 and 12 weeks of brace wearing and a week after brace removal)	Prospective cohort study	12–18 years old, affected by thoracic and previously untreated mild idiopathic scoliosis	NR	Risser localizer cast	NR	NR

Study ID	Population Baseline Characteristics Overall (Braced/Control): Cobb angle ± SD (thoracic)	Population Baseline Characteristics Overall: Cobb angle ± SD (lumbar)	Intervention Characteristics: Duration of bracing per day	Intervention Characteristics: Duration of bracing (months/years)	Time windows	Reported outcomes in study
Ran et al. 2016 [10]	(Sagittal) (22.2 ± 14.5/23.4 ± 15) (*n* = 60/177)	Not reported (NR)	17.2 h (10–22 h) (*n* = 60)	17.1 months (6–61 months) (*n* = 60)	Not applicable (NA)	FEV1(L), FVC(L)
Lin et al. 2022 [22]	14 to 97, average 46.58 (*n* = 168)	13 to 90 (average 41.7) (*n* = 168)	NR	NR	NA	FEV1 (% predicted), FVC(% predicted), FEV1/FVC ratio (%), Peak Expiratory Flow (PEF) (% predicted), Minute ventilation (% predicted), Tidal volume (% predicted), Respiratory frequency (/min), Ventilatory reserve (%)
Yurt et al. 2021 [12]	NR	NR	NR	8 months (*n* = 15)	First month/eighth month brace on/off (Short to long term effects)	FEV1 (L), FVC(L), FEV1/FVC ratio (%), Peak Expiratory Flow (L/S), Forced Expiratory Flow 25–75% (L/s), Maximal Inspiratory Pressure (cm H2O), Maximal Expiratory Pressure (cm H2O)
Refsum et al. 1990 [23]	NR	NR	23 h (*n* = 49)	3 weeks, 24 had further studies after 3 and 6 months with brace and after 1 day without brace (*n* = 49)	3 weeks after bracing (short term)	FEV1 (L), VC(L), FEV1/FVC ratio (%), Maximal Voluntary Ventilation (L/min), Total Lung Volume (L), Inspiratory Capacity (L), Functional Residual Capacity (L), Expiratory Reserve Volume (L), Residual Volume (L), Functional Residual Capacity/Total Lung Capacity (%), Residual Volume/Total Lung Capacity (%)
DiMaria et al. 2021 [24]	NR	NR	NR	10.09 ± 8.25 months (*n* = 11)	5 min after wearing the Sfozesco Thoracolumnar brace (immediate)	FEV1 (L), FVC(L), VC(L), FEV1/FVC ratio (%), Residual Volume (L), Total Lung Capacity (L), Intrathoracic Gas Volume (L), Maximal Voluntary Ventilation (L/min), Peak Expiratory Flow Rate(L/sec)
Yu et al. 2013 [25]	NR	NR	18.6 h, range 8–23 h (*n* = 70)	17.5 months (range 6–61 months) (*n* = 70)	NA	FEV1 (L), FVC(L)
Yagci et al. 2019 [11]	28.6 ± 7.0 (*n* = 27)	24.3 ± 6.5 (*n* = 27)	22.0 ± 1.0 h (*n* = 27)	1 month (*n* = 27)	1 month after bracing (with brace) (short term)	FEV1 (L), FVC(L), VC(L), FEV1/FVC ratio (%), Maximal Ventilator Volume (L/min), Peak Expiratory Flow, Forced Expiratory Flow 25–75, Maximal Inspiratory Pressure, Maximal Expiratory Pressure
Katsaris et al. 1999 [26]	NR	NR	NR	NR	Immediately in brace	FEV1 (L), VC (L), FEV1/FVC ratio (%), Residual Volume (L), Functional Residual Capacity (L), Total Lung Capacity (L), Static Lung Compliance, Specific Lung Compliance
Pehrsson et al. 2001 [27]	NR	NR	NR	NR	25 years after, in present follow up (long term)	FEV1 (L), VC (L)
Kennedy et al. 1989 [28]	NR	NR	NR	NR	Immediately in brace	VC(% predicted, without brace), Functional Residual Capacity (% predicted, without brace), Total Lung Capacity (% predicted, without brace)
Danielsson et al. 2015 [29]	NR	NR	22–24 h (n-68)	Until skeletal maturity, Risser 4 and a skeletal age 16 for girls and 18 for boys (*n* = 68)	NA	FEV1 (L), FVC(L), FEV1/FVC ratio (%)
Kennedy et al. 1987 [30]	NR	NR	NR	NR	Immediately in brace	FEV1 (L), FVC(L), Functional Residual Capacity (L), Total Lung Capacity (L)
Korovessis et al. 1996 [31]	28.7 ± 4.1 (*n* = 36)	26.5 ± 10.4 (*n* = 36)	20 h	NR	24 months after brace wearing (measurements taken both in brace and out of brace) (mid term)	FVC(L), VC(L), Forced Residual Capacity (FRC) (L), Residual Volume (RV) (L)
León-Morillas et al. 2021 [32]	NR	NR	NR	NR	NA	FEV1 (L), FVC(L), FEV1/FVC ratio (%), Peak Expiratory Flow (L/S), Maximum Inspiratory Pressure(cm H2O)
Margonato et al. 2005 [33]	Boys: 38 ± 5, Girls 42 ± 12. Total: 40 ± 9.12 (*n* = 8, 8, 16)	NR	NR	12 weeks (*n* = 16)	1 week and 12 weeks after brace wearing (short to mid term)	FVC (L/min), FEV1(L)

*NA* Not applicable, *NR* Not reported

* immediate/in brace = same day in brace; short term < = 3 months; mid term = 3–24 months; long term = > 24 months

**Table 4:** Sample size calculations

Study	Braced sample size	Control sample size	Total included for analysis	Irrelevant data (with explanation)/Withdrawals
Ran et al. 2016 [10]	60 (both genders)	177 (non braced)	237	No one withdrew from the study
Lin et al. 2022 [22]	51 (females only)	117 (females without a history of preoperative Boston brace treatment)	168	Originally, 176 patients signed the informed consent and agreed to participate. However, 8 dropped out
Yurt et al. 2021 [12]	15 (both genders)	0	15	Originally, there were 18 patients in the study. However, two stated that they did not use the brace regularly and one had an ongoing upper respiratory infection, hence the data was not recorded. (i.e. 3 dropouts)
Refsum et al. 1990 [23]	49 (females only)	0	49	No one withdrew from the study
Di Maraia et al. 2021	11(both genders)	0	11	No one withdrew from the study
Yu et al. 2013 [25]	70 (females only)	200 (females only, without preoperative brace)	270	No one withdrew from the study
Yagci et al. 2019 [11]	27 (females only)	0	27	No one withdrew from the study
Katsaris et al. 1999 [26]	15 (both genders)	0	15	No one withdrew from the study
Pehrsson et al. 2001 [27]	110 (both gender)	100 (age and gender matched, without scoliosis)	210	The study included 141 surgically treated patients which are irrelevant to the study Originally, the inclusion criteria were fulfilled by 283 patients(156 surgically treated, 127 braced), but only 141 and 110 patients were reinvestigated. 32 patients were not investigated
Kennedy et al. 1989 [28]	13 (both genders)	0	13	No one withdrew from the study
Danielsson et al. 2015 [29]	68 (both genders)	130 (age matched non scoliotic patients)	198	The study included 39 patients who underwent brace treatment and surgery and 17 who only underwent surgery. The data of these patients are excluded from the analysis
Kennedy et al. 1987 [30]	33 (both genders)	0	33	No one withdrew from the study
Korovessis et al. 1996 [31]	36 (both genders)	0	36	6 withdrew from the study
León- Morillas et al. 2021	4 (female patients)	7 (patients in both genders with scoliosis)	11	4 patients who underwent global posture reeducation were excluded from the analysis No one withdrew from the study
Margonato et al. 2005 [33]	16(both genders)	0	16	No one withdrew from the study
Total calculated	578	731	1309	

## Results

### Study selection and methodology quality

The initial search identified a total of 149 studies. After removal of duplicates, 126 studies underwent initial screening. A total of 20 studies underwent full-text screening by the reviewers, and finally, a total of 15 studies that satisfied the inclusion criteria were included in the review.

All studies were evaluated using the ROBIN-I (2016) score. Most of the studies are at moderate to serious risk of bias due to the presence of confounding factors (e.g. having non-AIS subjects as controls while measuring the difference with the braced AIS patients) and the methods of recruitment of participants in the study. In which, only one of the 15 studies indicated a low risk of bias, while nine of the 15 studies show moderate risk of bias. Four of the 15 studies show a serious risk of bias (Table 5) [19].

**Table 5:** Risk of bias: ROBINS-I (2016)

Study	Bias due to confounding	Bias in selection of participants into the study	Bias in classification of interventions	Bias due to deviations from intended interventions	Bias due to missing data	Bias in measurement of the outcome	Bias in selection of the reported result	Overall result
Ran et al. 2016 [10]	x	?	?	?	?	+	?	x
Lin et al. 2022 [22]	?	?	+	(not applicable due to the observative approach)	+	+	+	?
Yurt et al. 2021 [12]	?	+	+	?	+	+	+	+
Refsum et al. 1990 [23]	?	?	+	?	+	+	+	?
DiMaria et al. 2021 [24]	x	?	+	+	+	?	+	x
Yu et al. 2013 [25]	x	?	+	?	+	+	+	x
Yagci et al. 2019 [11]	?	+	+	?	+	+	+	?
Katsaris et al. 1999 [26]	x	?	+	+	+	?	+	?
Pehrsson et al. 2001 [27]	x	+	+	+	+	+	+	?
Kennedy et al. 1989 [28]	x	?	+	+	?	?	+	?
Danielsson et al. 2015 [29]	x	+	+	+	?	+	+	?
Kennedy et al. 1987 [30]	?	?	+	+	+	+	+	?
Korovessis et al. 1996 [31]	?	?	+	?	+	?	+	?
León-Morillas et al. 2021 [32]	x	x	?	?	+	?	?	x
Margonato et al. 2005 [33]	x	?	+	+	+	?	?	?

Scale: + low risk of bias,? moderate risk of bias, x serious risk of bias,! critical risk of bias

Three of these 15 included studies were comparative studies with the mean MINORS methodology score 17/24 ± 2 which indicates moderate quality research (Table 6). For the remaining 12 non-comparative studies, three of them scored above 13 in the MINORS methodology scores (14/16 ± 1), which stipulate good quality research, and the remaining nine studies have an average score of 11/16 ± 1, which indicate moderate research quality [20].

**Table 6:** Quality appraisal: MINORS scores

Study	A clearly stated aim	Inclusion of consecutive patients	Prospective collection of data	Endpoints appropriate to the aim of the study	Unbiased assessment of the study endpoint	Follow-up period appropriate to the aim of the study	Loss to follow up less than 5%	Prospective calculation of study size	An adequate control group	Contemporary groups	Baseline equivalence of groups	Adequate statistical analyses	MINORS score
Ran et al. 2016 [10]	low(2)	unclear(1)	high(0)	low(2)	low(2)	unclear(1)	low(2)	unclear(1)	low(2)	low(2)	low(2)	low(2)	19/24(high quality)
Lin et al. 2022 [22]	low(2)	unclear(1)	low(2)	low(2)	low(2)	unclear(1)	low(2)	high(0)					12/16 (moderate quality)
Yurt et al. 2021 [12]	low(2)	low(2)	low(2)	low(2)	low(2)	low(2)	high(0)	low(2)					14/16 (high quality)
Refsum et al. 1990 [23]	low(2)	unclear(1)	low(2)	low(2)	low(2)	low(2)	low(2)	low(2)					15/16(high quality)
DiMaria et al. 2021 [24]	low(2)	unclear(1)	low(2)	low(2)	low(2)	unclear(1)	low(2)	high(0)					12/16(moderate quality)
Yu et al. 2013 [25]	low(2)	unclear(1)	high(0)	low(2)	low(2)	high(0)	unclear(1)	high(0)	low(2)	low(2)	unclear(1)	low(2)	15/24 (moderate quality)
Yagci et al. 2019 [11]	low(2)	unclear(1)	low(2)	low(2)	low(2)	low(2)	low(2)	high(0)					13/16 (high quality)
Katsaris et al. 1999 [26]	low(2)	unclear(1)	low(2)	low(2)	low(2)	unclear(1)	low(2)	high(0)					12/16 (moderate quality)
Pehrsson et al. 2001 [27]	low(2)	low(2)	high(0)	low(2)	low(2)	low(2)	high(0)	high(0)	low(2)	low(2)	unclear(1)	low(2)	17/24 (moderate quality)
Kennedy et al. 1989 [28]	low(2)	high(0)	low(2)	low(2)	low(2)	unclear(1)	low(2)	high(0)					11/16 (moderate quality)
Danielsson et al. 2015 [29]	low(2)	low(2)	high(0)	low(2)	low(2)	low(2)	high(0)	high(0)					10/16(moderate quality)
Kennedy et al. 1987 [30]	low(2)	unclear(1)	low(2)	low(2)	low(2)	unclear(1)	low(2)	high(0)					12/16 (moderate quality)
Korovessis et al. 1996 [31]	low(2)	unclear(1)	low(2)	low(2)	low(2)	low(2)	high(0)	high(0)					11/16 (moderate quality)
León-Morillas et al. 2021 [32]	low(2)	low(2)	high(0)	low(2)	low(2)	unclear(1)	low(2)	high(0)					11/16 (moderate quality)
Margonato et al. 2005 [33]	low(2)	unclear(1)	low(2)	low(2)	unclear(1)	low(2)	low(2)	high (0)					12/16 (moderate quality)

### Study characteristics

The 15 papers included 1 retrospective cohort study, 9 prospective studies, 1 case series, 1 retrospective case series, 2 longitudinal observational follow-up studies and 1 case–control study. (Table 3) The publishing years of the included papers were between 1987 to 2022. The studies included in the analysis had varying sample sizes, ranging from 11 to 270 cases of idiopathic scoliosis in adolescent populations, with a total of 578 cases with adolescent idiopathic scoliosis and 731 controls. The age of the study participants ranged between 10 and 18.4 years old. All the studies specified the types of braces included and four different types of braces which were reported in the studies are: Cheneau brace, Sforzesco, Boston and Milwaukee.

### Pulmonary function

All studies except Korovessis et al. [31] and Kennedy et al. [28] included FEV1 in their measurement of pulmonary function. A wide range of pulmonary function parameters were acquired in these 15 studies, which include: respiratory muscle strength, FVC, FEV1/FVC ratio, Peak Expiratory Flow/Rate, Minute Ventilation, Tidal Volume, Respiratory Frequency, Ventilatory Reserve, Peak Expiratory Flow, Forced Expiratory Flow 25–75%, Maximal Inspiratory Pressure, Maximal Expiratory Pressure, Maximal Voluntary Ventilation, Total Lung Volume/Capacity, Inspiratory Capacity, Functional Residual Capacity, Residual Capacity/Volume, Expiratory Reserve Volume, Functional Residual Capacity/Total Lung Capacity, Residual Volume/Total Lung Capacity, Intrathoracic Gas Volume, Peak Expiratory Flow Rate etc.

The data extracted from the studies are listed in Tables 7, 8, 9, 10.

**Table 7:** Summary of the data regarding FEV1 values extracted from the studies (*n* = 13)

				**Beginning of Study/Control**				**End of Study/Braced**				
**FEV1**	**unit**	**Measurement: before**	**Measurement: end result**	**mean ± SD**	**95% confidence interval**	**min–max**	***p*** value	**mean ± SD**	**95% confidence interval**	**min–max**	***p*** value	**Effect size via Cohen’s d approximation (standard mean difference)**
Danielsson et al. 2015 [29]	% predicted	control (without scoliosis)	brace treatment group (brace off)	107.1 ± 13.8		57.0–144.0	< 0.0001	85.5 ± 12.3		55.9–122.0	< 0.0001	1.68 (large)
Yu et al. 2013 [25]	L/% predicted	control (without brace treatment)	brace treatment group	2.48 ± 0.5/91.1% ± 16.9%			% predicted *p* < 0.05	2.37 ± 0.6/85.7% ± 18.9%				0.209(small)/0.293 (small)
Ran et al. 2016 [10]	L	control (without brace treatment)	brace treatment group	2.52 ± 0.56			< 0.0001, power 0.981	2.24 ± 0.34			< 0.0001, power 0.981	0.654 (medium)
Lin et al. 2022 [22]	% predicted	no brace treatment	brace treatment group	89.0 ± 16.7			0.739	89.9 ± 13.4			0.739	0.0570(small)
León-Morillas et al. 2021 [32]	L	no intervention	brace treatment group	2.82 ± 0.85			0.51	1.98 ± 0.87			0.51	0.980 (large)
Kennedy et al. 1987 [30]	L	brace off	brace on	2.4 ± 0.5			< 0.001	2 ± 0.5			< 0.001	0.8 (large)
Yurt et al. 2021 [12]	L	brace off (18 h off brace before measurement) (First month/eighth month)	brace on (first month/eighth month)	2.5 ± 0.4(first month, brace off), 2.4 ± 0.4 (eighth month, brace off)	2.2–2.7/2.1–2.7		< 0.001	2.0 ± 0.4(first month, brace off), 1.9 ± 0.4 (eighth month, brace off)	1.8–2.3/1.8–2.2		< 0.001	1.25(large)/1.25 (large)
Katsaris et al. 1999 [26]	L	brace off	immediately after brace on	2.5 ± 0.41		1.6–3.1	0.2029	2.3 ± 0.43		1.4–2.9	0.2029	0.476(medium)
DiMaria et al. 2021 [24]	L	Resting breathing, brace off	5 min after wearing Sforzesco Thoracolumbar brace	2.44(0.25 standard error of mean)			< 0.05	1.92(0.19 standard error of mean)			< 0.05	NA
Refsum et al. 1990 [23]	L	brace off (pre-treatment)	3 weeks after bracing (with braced)	2.57 ± 0.06			< 0.001	2.05 ± 0.06			< 0.001	8.67(large)
Yagci et al. 2019 [10]	L	brace off (unbraced)	1 month after bracing (with brace)	2.3 ± 0.6			< 0.001	1.8 ± 0.7			< 0.001	0.767 (large)
Margonato et al. 2005 [33]	L	Exercising before bracing	Exercising brace off (1 week/12 weeks/1 week after brace removal)	3.9 ± 1.0 (males), 2.8 ± 0.3 (females)			< 0.05	week 1: 2.5 ± 1.2(males, *p* < 0.05), 2.0 ± 0.6(females, *p* < 0.05) Week 12: 3.4 ± 1.0(males, *p* > 0.05), 2.6 ± 0.35 (females, *p* > 0.05) 1 week after removal of brace: 3.7 ± 1.0(males, *p* > 0.05), 2.8 ± 0.1(females, *p* > 0.05)				Week 1: males: 1.27 (large)/1.69 (large) Week 12: males 0.5 (medium)/females 0.614 (medium) 1 week after removal of brace: males 0.2 (small)/females 0 (small)
Pehrsson et al. 2001 [27]	L	Before treatment	25 years after present follow up	2.9 ± 0.5		1.7–4.1	< 0.01	3.1 ± 0.5		1.0–4.4	< 0.01	0.4 (medium)

^*^ immediate/in brace = same day in brace; short term < = 3 months; mid-term = 3–24 months; long term = > 24 months

**Table 8:** Summary of the data regarding FVC values extracted from the studies (*n* = 11)

				**Beginning of Study/Control**				**End of Study/Braced**				
**FVC**	**unit**	**Measurement: before**	**Measurement: end result**	**mean ± SD**	**95% confidence interval**	**min–max**	***p*** value	**mean ± SD**	**95% confidence interval**	**min–max**	***p*** value	**Effect size via Cohen’s d approximation (standard mean difference)**
Danielsson et al. 2015 [29]	% predicted	control (without scoliosis)	brace treatment group (brace off)	102.4 ± 11.7		70.9–127.8	< 0.0001	89.9 ± 12.1		55.6–114.0	< 0.0001	1.06(large)
Yu et al. 2013 [25]	L/% predicted	control (without brace treatment)	brace treatment group	2.72 ± 0.6/85.2% ± 16.5%			% predicted *p* < 0.05	2.61 ± 0.6/80.6% ± 17.0%				0.183 (small)/0.277 (small)
Ran et al. 2016 [10]	L	control (without brace treatment)	brace treatment group	2.34 ± 0.54			0.9019, power 0.052	2.35 ± 0.55			0.9019, power 0.052	0.0184 (small)
Lin et al. 2022 [22]	% predicted	no brace treatment	brace treatment group	88.8 ± 16.5			0.917	89.1 ± 13.3			0.917	0.0192(small)
León-Morillas et al. 2021 [32]	L	no intervention	brace treatment	3.57 ± 0.78			0.146	2.77 ± 0.37			0.146	1.19 (large)
Yurt et al. 2021 [12]	L	brace off (18 h off brace before measurement) (First month/eighth month)	brace on (first month/eighth month)	2.9 ± 0.5(first month, brace off), 2.8 ± 0.4 (eighth month, brace off)	2.5–3.2/2.4–3.1		< 0.001	2.4 ± 0.5(first month, brace off), 2.2 ± 0.4 (eighth month, brace off)	2.1–2.7/1.9–2.5		< 0.001	1 (large)/1.5 (large)
Kennedy et al. 1987 [30]	L	brace off	brace on	2.8 ± 0.6			< 0.001	2.3 ± 0.6			< 0.001	0.833(large)
DiMaria et al. 2021 [24]	L	Resting breathing, brace off	5 min after wearing Sforzesco Thoracolumbar brace	2.77(standard error of mean 0.88)			< 0.05	2.25(standard error of mean 0.68)			< 0.05	NA
Korovessis et al. 1996 [31]	L	beginning of study (without brace/with brace)	24 months after brace wearing (no brace/braced)	3.5 ± 0.5/2.45 ± 0.5			0.03	3.7 ± 0.5/2.7 ± 0.5			0.03	0.4 (medium)/0.5 (medium)
Yagci et al. 2019 [11]	L	brace off	1 month after bracing (with brace)	3.1 ± 0.8			< 0.001	2.4 ± 0.9			< 0.001	0.822 (large)
Margonato et al. 2005 [33]	L/min	Exercising before bracing	exercising while brace off (1 week after start of bracing/12 weeks after start of bracing/1 week after brace removal)	4.3 ± 1.0 (males), 3.0 ± 0.4 (females)			< 0.05	week 1: 2.8 ± 1.15(males, *p* < 0.05), 2.1 ± 0.8(females, *p* < 0.05) Week 12: 3.9 ± 1.0(males, *p* < 0.05), 2.8 ± 0.4 (females, *p* > 0.05) 1 week after removal of brace: 4.1 ± 0.9(males, *p* > 0.05), 3.1 ± 0.4(females, *p* > 0.05)				week 1 males: 1.39(large), females: 1.42 (large) Week 12 males: 0.4 (medium), females: 0.5 (medium) 1 week after removal of brace: males: 0.210 (small), females, 0.25 (small)

^*^ immediate/in brace = same day in brace; short term < = 3 months; mid term = 3–24 months; long term = > 24 months

**Table 9:** Summary of the data regarding VC values extracted from the studies (*n* = 7)

			**Beginning of Study/Control**			**End of Study/Braced**			
**VC (L)**	**Measurement: before**	**Measurement: end result**	**Mean ± SD**	**Min–Max**	***p*** value	**Mean ± SD**	**Min–Max**	***p*** value	**Effect size via Cohen’s d approximation (standard mean difference)**
Kennedy et al. 1989 [28]	Brace off	Immediately after brace on	2.8 ± 0.721		< 0.01	2.4 ± 0.721		< 0.01	0.555(medium)
Katsaris et al. 1999 [26]	Brace off	Immediately after brace on	2.8 ± 0.56	1.7–3.7	0.1363	2.5 ± 0.51	1.6–3.2	0.1363	0.560 (medium)
DiMaria et al. 2021 [24]	Resting breathing, brace off	5 min after wearing Sforzesco Thoracolumbar brace	2.63 (Standard error of mean 0.24)		< 0.05	2.06(standard error of mean 0.17)		< 0.05	NA
Refsum et al. 1990 [23]	Brace off	3 weeks after bracing (with brace)	2.98 ± 0.07		< 0.001	2.38 ± 0.06		< 0.001	9.20 (large)
Yagci et al. 2019 [11]	Brace off	1 month after bracing (with brace)	2.6 ± 0.9		< 0.001	2.1 ± 0.8		< 0.001	0.587 (medium)
Korovessis et al. 1996 [31]	Beginning of study (without brace/with brace)	24 months after brace wearing (no brace/braced)	3.4 ± 0.5/2.5 ± 0.5		0.02	3.7 ± 0.5/2.7 ± 0.5		0.02	0.6 (medium)/ 0.4 (medium)
Pehrsson et al. 2001 [27]	Before treatment	25 years after present follow up	3.4 ± 0.6	2.1–4.7	< 0.001	3.8 ± 0.7	2.4–5.8	< 0.001	0.614(medium)

^*^immediate/in brace = same day in brace; short term < = 3 months; mid term = 3–24 months; long term = > 24 months

**Table 10:** Summary of the data regarding FEV1/FVC Ratio extracted from the papers (*n* = 7)

			**Beginning of Study/Control**				**End of Study/Braced**				
**FEV1/FVC (%)**	**Measurement: before**	**Measurement: end result**	**Mean ± SD**	**95% confidence interval**	**Min–Max**	***p*** value	**Mean ± SD**	**95% confidence interval**	**Min–Max**	***p*** value	**Effect size via Cohen’s d approximation** **(standard mean difference)**
Lin et al. 2022	No brace treatment	Brace treatment group	84.1 ± 10.8			0.341	85.6 ± 5.03			0.341	0.376 (small)
Katsaris et al. 1999 [26]	Brace off	Immediately after brace on	89.5 ± 5.85		80–100	0.3256	91.8 ± 6.71		78–100	0.3256	0.365 (small)
Danielsson et al. 2015 [29]	Control (without scoliosis)	Brace treatment group (brace off)	79.5 ± 6.9		45.9–99.5	0.0003	77.7 ± 5.8		62.5–89.1	0.43	0.275 (small)
León-Morillas et al. 2021 [32]	No intervention	Brace treatment group	93.29 ± 17.78			0.239	80.75 ± 30.72			0.239	0.547 (medium)
DiMaria et al. 2021 [24]	Resting breathing, brace off	5 min after wearing Sforzesco Thoracolumbar brace	88(standard error of mea*n* = 2)			not significant	85(standard error of mea*n* = 2)			not significant	NA
Yagci et al. 2019 [11]	Brace off	1 month after bracing (with brace)	77.4 ± 15.2			0.407	74.3 ± 11.9			0.407	0.227 (small)
Yurt et al. 2021 [12]	Brace off (18 h off brace before measurement) (First month/eighth month)	Brace on (first month/eighth month)	88.0 ± 5.0(brace-off at 1 st month), 86.8 ± 6.8(brace-off at 8th month)	84.0–91.1/81.9–91.6		0.621/0.297	86.0 ± 5.3(brace-off at 1 st month), 85.7 ± 8.2 (brace-off at 8th month)	82.9–89.1/79.8–91.5		0.621/0.297	0.388 (medium)/0.146 (small)

^*^immediate/in brace = same day in brace; short term < = 3 months; mid term = 3–24 months; long term = > 24 months

There are 13 studies detailing the FEV1 of the braced patients. Results shown in Table 7 generally indicate that the FEV1 is lower when braced compared to unbraced. 10 studies are statistically significant.

#### Short-term effects of bracing on FEV1

Danielsson et al. [29] showed that the FEV1 is lower in AIS patients when the brace is off, compared to those without scoliosis. The percentage prediction of FEV1 was 107.1% ± 13.8% in non-scoliotic controls compared to the level of 85.5% ± 12.3% in scoliotic patients out of brace. The result is statistically and practically significant (*p* < 0.0001, standard mean difference 1.68).

Kennedy et al. [30], Katsaris et al. [26] and DiMaria et al. [24] compared the FEV1 of the AIS patients when they are on brace and off brace. The results of Katsaris et al. [26] are not statistically significant. However, Kennedy et al. [30] reported statistically significant results (*p* < 0.001) and showed that there is a significant drop of FEV1 after the brace is on (2.4 ± 0.5L brace off to 2.0 ± 0.5L brace on, standard mean difference 0.8). DiMaria et al. [24] also reported statistically significant results (*p* < 0.05) and a drop from 2.44L when off brace to 1.92L after wearing the brace. This implies the restrictive effect of the braces would cause a decrease in the FEV1, rather than improve it due to the correction of posture.

Yu et al. [25], Ran et al. [10], Lin et al. (2022) and León-Morillas et al. [32] compared scoliotic patients with brace interventions and another group without brace. However, the results from Lin et al. (2022) and León-Morillas et al. [32] are not statistically significant (*p* > 0.05). Yu et al. [25] and Ran et al. [10] show a decrease in FEV1 between the controls and the braced group, and the results are statistically significant (*p* < 0.05). However, there are variable differences between the groups, as Yu et al. [25] only reported a small difference(standard mean difference 0.209/0.293), while Ran et al. [10] recorded a meaningful relationship between bracing and pulmonary function(2.52 ± 0.56L in scoliotic patients without brace treatment to 2.24 ± 0.34L when undergoing brace treatment, standard mean difference 0.654). Hence, we could draw the conclusion that bracing would decrease FEV1, however, the clinical significance of the change is yet to be seen.

Refsum et al. [23] and Yagci et al. [11] showed the short-term change in FEV1 measured while brace on compared to unbraced levels (short term < = 3 months). All of the studies are statistically significant(*p* < 0.05). Within weeks to a month with bracing, there is a significant decrease in FEV1 compared to the level before bracing (2.57 ± 0.06L before bracing to 2.05 ± 0.06 in 3 weeks:, standard mean difference 8.67) and unbraced levels (2.3 ± 0.6L to 1.8 ± 0.7 in 1 month, standard mean difference 0.767). This reinforced that in the short term, bracing continues to decrease the FEV1 level, and the respiratory function could not increase back to the state of unbraced/prebracing.

Margonato et al. [33] also measured the short-term changes of the effects on a patient’s exercise capabilities by comparing the pre-bracing exercise FEV1 to the level a week later off brace. The results are statistically significant for both males and females (*p* < 0.05). There is a significant decrease in FEV1 in both genders, decreasing from 3.9 ± 1.0L to 2.5 ± 1.2L for males (standard mean difference: 1.27) and 2.8 ± 0.3L to 2.0 ± 0.6L (standard mean difference: 1.69) for females. In the short term, bracing decreases the FEV1 and the exercise ability of all patients, even when the brace is removed.

#### Long-term effects of bracing on FEV1

Margonato et al. [33], Yurt et al. [12], and Pehrsson et al. [27] studied the long-term effects of bracing on whether bracing by investigating whether bracing would improve or impair the respiratory function in the long term. Margonato et al. [33] and Yurt et al. [12] studied the carryover effects up to 8 months after bracing. However, the impact of 3 months of brace wearing and a week of removal of the brace on FEV1 during exercise is rendered statistically insignificant as the p value is > 0.05 in Margonato et al. [33]. Yurt et al. [12] demonstrated that the FEV1 would decrease slightly under long-term bracing (first month 2.5 ± 0.4L brace-off to 2.4 ± 0.4L brace-off in the eighth month, first month 2.0 ± 0.4L brace-on to 1.9 ± 0.4L brace-on in the eighth month, p < 0.001). The change is clinically significant as the effect size is large (1.25 for both measurements). It may be due to the prolonged restrictive effect of the brace, which results in impairment in the development of respiratory muscles.

In a 25-year follow-up, Pehrsson et. al [27] demonstrated that the FEV1 of the patients before brace treatment and 25 years after brace treatment would increase (2.9 ± 0.5L before brace treatment to 3.1 ± 0.5L after 25 years, *p* < 0.01, standard mean difference 0.4). However, it is crucial to note that the growth of the patients could cause the apparent increase in FEV1, and the % predicted remained lower than that of the same group of adults without scoliosis. (91% ± 16% in braced patients and 104% ± 13% in normal matched adults, *p* < 0.001).

Eleven studies reported on their findings on the FVC. The data shown in Table 8 generally shows that the FVC is lower when braced compared to unbraced. 8 studies are statistically significant.

#### Short-term effects of bracing on FVC

Danielsson et al.[29] showed that in controls without scoliosis and compared to brace-treated groups under the brace-off measurement condition, the % predicted FVC would decrease significantly, from 102.4 ± 11.7% to 89.9 ± 12.1%, *p* < 0.0001, standard mean difference 1.06. Populations of AIS patients who require bracing have an innate reduction of their FVC.

Yu et al. [25], Ran et al. [10], Lin et al. (2022) and León-Morillas et al. [32] showed that those AIS patients without brace treatment would do better compared to the brace treatment group. However, the results from Ran et al. [10], Lin et al. (2022) and León-Morillas et al. [32] are not statistically significant (*p* > 0.05). Even so, Yu et al. [25] showed there is just a small clinically significant decrease (2.72 ± 0.6L/85.2% ± 16.5% in scoliotic patients without brace treatment to 2.61 ± 0.6L/80.6% ± 17.0% of those undergoing brace treatment, standard mean difference 0.183/0.277). Therefore, there is only a minimal decrease in FVC compared to those unbraced subjects.

Kennedy et al. [30] and DiMaria et al. [24] discussed the immediate effect of bracing on reducing the FVC, for which the values drop from 2.8 ± 0.6L brace off to 2.3 ± 0.6L brace on, *p* < 0.001, with a significant effect size of 0.833, and 2.77L (SEM 0.88) to 2.25L(SEM 0.68), *p* < 0.05, respectively. The immediate restrictive effect of bracing is prominently reflected in the decrease in FVC. Yagci et al. [11] also displayed that there is a statistically significant decrease(*p* < 0.001) in FVC when comparing braced and unbraced values after a month of bracing. The FVC dropped from 3.1 ± 0.8L to 2.4 ± 0.9L, with a standardized mean difference of 0.822. This means that even over time, the restrictive effects of bracing remain prominent and decrease the FVC of AIS patients.

Margonato et al. [33] had shown a decrease in FVC during exercise after 1 week of bracing. The decrease is statistically significant (*p* < 0.05). There is a drop of FVC from 4.3 ± 1.0L when exercising pre-braced to 2.8 ± 1.15L after a week of bracing for males, and the drop from 3.0 ± 0.4L when exercising pre-braced to 2.1 ± 0.8L a week after bracing in females. The decrease is clinically significant with a large standard mean difference, meaning that the drop is substantial and affects the AIS patients’ respiratory function.

#### Long-term effects of bracing on FVC

Yurt et al.[12] and Korovessis et al. [31] showed statistically significant results in months-long bracing. FVC of braced patients decreased when they wore the brace compared to the baseline brace-off measurements. Yurt et al. [12] showed that the FVC would decrease slightly under long-term bracing (first month 2.9 ± 0.5L brace off to 2.4 ± 0.5L brace off in the eighth month, first month 2.8 ± 0.4L brace on to 2.2 ± 0.4L brace on in the eighth month, p < 0.001). The effect size is also large(1 and 1.5 in the first and eighth month respectively), indicating that the difference is clinically significant. Korovessis et al. [31] compared the effect of brace wearing over two years. It is interesting that there is a paradoxical increase in FVC after 24 months of brace-wearing measured both with and without brace. The result is interesting, indicating possible reversibility and improvement in FVC. The height and growth of the patients would also need to be accounted for to determine whether the improvement is truly due to bracing or purely attributed to growth.

Margonato et al. [33] has variable data regarding the FVC after 12 weeks of bracing. The change in FVC during exercise in males (off brace) is statistically significant, yet the remaining data, i.e. the change in FVC in females and the FVC 1 week after brace removal, is statistically insignificant. In males, the decrease in FVC after 12 weeks of bracing has moderate clinical value, with the standard mean difference being 0.4 (4.3 ± 1.0L to 3.9 ± 1.0L). Hence, we can conclude that there is a decrease in FVC in males, but the reason why the decrease is not reflected in females remains unknown.

There are 7 studies reporting on their findings on the VC. The data shown in Table 9 generally show that the VC is lower under braced conditions when compared to unbraced conditions, with 6 studies revealing a statistically significant result.

#### Short-term effects of bracing on VC

Kennedy et al. [28] and DiMaria et al. [24] measured the immediate effect on VC after bracing. They show a statistically significant decrease, from 2.8 ± 0.721L to 2.4 ± 0.721L, *p* < 0.01 and 2.63L (SEM 0.24L) to 2.06 (SEM 0.17L) *p* < 0.05. The decrease that Kennedy et al.[28] showed is also moderately clinically significant with a medium effect size.

Refsum et al. [23] and Yagci et al. [11] illustrated the short term change in VC after 3 to 4 weeks of bracing. Refsum et al. [23] and Yagci et al. [11] showed a decrease in VC after bracing, from 2.98 ± 0.07L to 2.38 ± 0.06L (*p* < 0.001) and 2.6 ± 0.9L to 2.1 ± 0.8L(*p* < 0.001). In particular, the decrease in VC in Refsum et al. [23]’s study when comparing pre bracing values to weeks after bracing (with bracing) is large, indicating a substantial difference pre-bracing and during bracing.

#### Long-Term Effects of Bracing on VC

Korovessis et al.[31] and Pehrsson et al. [27] showed the long-term effects of bracing. (24 months, and 25 years respectively) Both studies are statistically significant(p < 0.05) and have moderate clinical significance.

Korovessis et al.[31] showed an interesting trend, that there is an increase in VC from the value measured at the beginning of the study to the 24th month. For those with AIS, the data at the beginning of the study, 3.4 ± 0.5L (brace off) to 3.7 ± 0.5L (brace off) in the 24th month. When the brace is on, Korovessis et al. measured an increase from the beginning of the study, 2.5 ± 0.5L brace on to 2.7 ± 0.5L in the 24th month, *p* = 0.02. This indicates a possible reversal of the bracing effect. However, further studies are needed to confirm whether the change is attributed to growth or bracing.

Pehrsson et al. [27] showed an increase in the VC from 3.4 ± 0.6 to 3.8 ± 0.7, *p* < 0.001, before treatment to 10 years follow-up. However, other factors, such as growth in height and lung capacity, had not been considered. Compared to the age-matched controls without scoliosis, the % predicted VC is still lower than normal, being 89 ± 13% (range 56–127%), in contrast to the controls being 103 ± 11%. While it is natural for scoliotic patients to have a lower VC compared to the general population, a more detailed analysis is required to determine whether bracing is beneficial as a treatment therapy in the long-term for improving VC.

There are 7 studies reporting on their findings on the FEV1/FVC ratio. The data shown in Table 10 generally shows that the FEV1/FVC ratio is lower when braced compared to unbraced. No study is statistically significant.

#### Effect of bracing on FEV1/FVC ratio

Lin et al. [22] and Katsaris et al. [26] showed that there is an increase in FEV1/FVC ratio, yet others, e.g. Yagci et al. [11] and Yurt et al. [12], showed that there is a decrease in FEV1/FVC ratio. However, most statistics in the study are statistically insignificant, as the *p* > 0.05, including Danielsson et al. [29]. Hence, the effect of bracing on the FEV/FVC ratio in both the short term and the long run was inconclusive.

## Discussion

The results provide a comprehensive understanding of the impacts of bracing on pulmonary function in AIS patients. 15 papers of variable quality are found and analyzed.

### Short-term pulmonary function affected by bracing with FEV1, FVC, VC, FEV1/FVC ratio measurements

Going through the studies, FEV1, FVC and VC underwent significant changes when immediately braced, and the reduction of pulmonary function persists even after wearing the braces for months or years. The immediate drop of FEV1 and FVC is clinically and statistically significant when comparing brace on and brace off in scoliotic patients, yet there are smaller clinical significance in the FEV1 when comparing scoliotic patients with brace treatment and without brace treatment. Wearing the brace by itself was associated with a decrease in FEV1, however, when the brace was off, the FEV1 value did not differ between those in the treatment group and non-treatment group. Clinically and statistically significant findings could be summarized in the decrease in FVC comparing brace on to brace off. The braced FVC and VC remain lower than unbraced values and prebracing values even after a month of bracing, signifying that the restrictive effects of bracing are still prominent.

In the short term, the FEV1 and FVC when the brace is removed after several weeks of bracing are also decreased compared to pre-bracing levels. The recorded VC is revealed to be lower after weeks of brace wearing compared to brace off levels. The exercise FEV1 and FVC were also observed to be lower.

Statistically significant decreases in FEV1, FVC and VC are found, indicating the immediate decrease in respiratory function of the AIS patients. FEV1/FVC ratio did not yield statistically significant changes, but combined with the decrease in FEV1 and FVC indicated a restrictive ventilatory pattern.

### Long-term pulmonary function affected by bracing with FEV1, FVC, VC, FEV1/FVC ratio measurements

Upon follow-up evaluation performed months after bracing, the braced state was still correlated with decreased FEV1, FVC and VC. Again, no statistically significant result could be found in the FEV1/FVC ratio, indicating a prolonged restrictive respiratory pattern. However, it is interesting to note that Yurt et al. [12] showed that after months of brace wearing, FVC and FEV1 decreased slightly even after the brace removal. There is an association between the decrease of baseline function of the patients and the use of the braces in the long term.

For follow-up years after the bracing regime had been completed, we could note the improvement of the pulmonary function of the patients, especially in FEV1 and VC. Further testing can be used to confirm the results, and the control of the confounding factors, e.g. respiratory capacity increase of the patient and height growth, or baseline difference between non-scoliotic controls and braced AIS patients, had to be done to validate the results.

### Potential direction: exercise to improve respiratory function

It is clinically popular to incorporate exercise with bracing to improve the respiratory function of AIS patients. In particular, aerobic exercise and Schroth-specific exercises are frequently implemented. While this review focuses on the relationship between pulmonary function and bracing, multiple studies retrieved in the reviewing process emphasized the effectiveness of exercise on improving the respiratory function of AIS patients. We would like to give a summary of those research papers on the importance of exercise, yet further analysis is required to quantify the improvement and to draw a conclusion on the effectiveness of the intervention.

Athanasopoulos et al. [34] discovered that exercise would improve the pulmonary function of AIS patients. Aerobic training maintained the VC and FEV1and significantly improved the patient’s FVC and FEV1/FVC ratio. The control group experienced a decline in VC and FVC during the two-month period. The aerobic work performance capability increased 48.1% (*P* < 0.01) in the training group, while it decreased 9.2% (*P* < 0.01) in the control group.

Stein et al. [35] and Badowska et al. [36] measured the impact of Schroth-specific exercises on pulmonary function. In Badowska et al. [36], before the exercises, the restrictive disorder occurred in 61.5% of patients, while after their Schroth-specific exercise, the disorder prevalence dropped to 34.6% of that cohort of patients, indicating a positive effect of the scoliosis-specific exercise therapy on improving the volumetric pulmonary function for AIS. This relationship may result from the improvement in the values of VC and FVC parameters after the exercises. According to Stein et al. [35], there were significant improvements for FVC + 3.99%, FEV1 + 2.36% after the patients trained in Schroth-specific exercises for the first rehabilitation stay, and FVC continued to improve after the second and third rehabilitation stays.

### Limitation of the literature search

Fifteen relevant research papers were included in this review, but most of them were case–control studies, case series, and cohort studies. There are no randomized control trials (RCTs) retrieved from our search. The sample sizes of the included studies were small; hence, statistically significant results are difficult to obtain. Bracing data on the duration of bracing per day in hours and the total duration in months to years are also missing. Also, the progression of the scoliotic curvature was not measured in the studies after a few months of bracing, potentially contributing to the decreasing pulmonary function. Many studies did not specify the enrollment of consecutive patients, did not eliminate major variables and confounding factors, and retrospective data; the quality of the studies is highly variable and heterogeneous, and a meta-analysis was therefore not performed in this systematic review.

Furthermore, while translations of the relevant papers are included, articles in languages other than English are excluded. Other limitations regarding the unity of age, gender, and compliance of the subjects, as well as the heterogeneity of type and severity of the spinal curvatures of the patients obtained from the articles, vary. Further studies are suggested to provide a more holistic picture of the long-term effect of bracing on AIS patients.

## Conclusion

Existing research indicates that bracing has an immediate restrictive effect on pulmonary function. This immediate decrease in pulmonary function persists when braced for several months and measured with the brace on. The function continues to decrease after longer periods of bracing in both in-brace and out-brace measurements. In the long-term, years after the completion of the bracing regime, while the adults still exhibit a lower pulmonary function compared with the non-scoliotic controls, their pulmonary function improved significantly compared with the pre-bracing level.

## Data Availability

The datasets used and/or analysed during the current study are available from the corresponding author on reasonable request.

## Abbreviations

AIS: Adolescent Idiopathic Scoliosis
FEV1: Forced Expiratory Volume in 1 Second
FEV1/FVC ratio: The ratio between Forced Expiratory Volume in 1 Second and Forced Vital Capacity
FVC: Forced Vital Capacity
MINORS methodology score: Methodological Index for Non-Randomized Studies
PEF: Peak Expiratory Flow
PICO: Patient, Intervention, Comparison and Outcome
PRISMA: Preferred Reporting Items for Systematic Reviews and Meta-Analyses
ROBINS-I: Risk of Bias In Non-randomized Studies—of Interventions
VC: Vital Capacity
